# The complete chloroplast genome sequence of *Rheum tanguticum*, an endangered Chinese medicinal plant (*Polygonaceae*)

**DOI:** 10.1080/23802359.2019.1688722

**Published:** 2019-11-13

**Authors:** Xiaowei Huo, Hao Wei, Jing Gao, Yonggang Yan, Gang Zhang, Mengmeng Liu

**Affiliations:** a Key Laboratory of Pharmaceutical Quality Control of Hebei Province, College of Pharmaceutical Science, Hebei University, Baoding, PR China;; b College of Pharmacy and Shaanxi Qinling Application Development and Engineering Center of Chinese Herbal Medicine, Shaanxi University of Chinese Medicine, Xi'an, PR China;; c College of Traditional Chinese Medicine, Hebei University, Baoding, PR China;; d Institute of Bioinformatics and Medical Engineering, School of Electrical and Information Engineering, Jiangsu University of Technology, Changzhou, PR China

**Keywords:** *Rheum tanguticum*, chloroplast genome, conservation, phylogenomics

## Abstract

*Rheum tanguticum* is a valuable medicinal plant endemic to the Qinghai-Tibetan Plateau. It has been listed classified under the IUCN Red List categories of Vulnerable due to the low reproductive rate and heavy exploitation. In this study, the complete chloroplast (cp) genome of *R. tanguticum* has been assembled using data from the whole-genome Illumina sequencing. The cp genome is 161,515 bp in size and contains two inverted repeat regions of 30,823 bp each, which is separated by a large single-copy region of 86,675 bp and a small single-copy region of 13,194 bp. The cp genome contains 87 protein-coding genes, 37 tRNA genes, and 8 rRNA genes. Phylogenetic analysis revealed that the cp genome of *R. tanguticum* was closely related to that of the *R. palmatum*.

*Rheum tanguticum* belonging to the family Polygonaceae is a traditional Chinese medicine that has been widely used as a purgative and anti-inflammatory agent for 2000 years (Chen et al. 2009). It has been listed as ‘Vulnerable’ by the IUCN due to the low reproductive rate and heavy exploitation (Zhang et al. 2014). In this study, we aimed to assemble and characterize the chloroplast (cp) genome of *R. tanguticum* to contribute to the effective conservation of this vulnerable plant.

Total genomic DNA was extracted from fresh leaves of an individual plant collected from Qinghai-Tibetan Plateau (N33°45′3.83′′, E99°39′1.28′′) using EASYspin plus Total DNA Isolation Kit (Aidlab, Beijing, China) following the manufacturer’s instructions. A voucher specimen (No. TGTDH-03-0418) was also deposited at the herbarium of Hebei University, Baoding, China. An amount of 5 μg genomic DNA was used for high throughput sequencing with the Illumina Hiseq 2500 platform by Majorbio Biotechnologies Inc. (Shanghai, China). Approximately 1.5 GB of raw data were generated through pair-end 150 bp sequencing. CLC Genomics Workbench version 9 (CLC Bio, Aarhus, Denmark) was used to remove the adapter sequences with the default parameters set. The clean data were fed into the MITObim version 1.8 (Hahn et al. 2013) for assembly with *R. palmatum* (GenBank: KR816224) as the reference sequence. The cp genome was annotated using standard settings in the online program DOGMA (Lohse et al. 2013).

The cp genome of *R. tanguticum* (GenBank: MK674897) is 161,515 bp in length, consisting of a pair of inverted repeat regions of 30,823 bp each, a large single-copy region of 86,675 bp, and a small single-copy region of 13,194 bp. The overall GC content of the cp genome was 37.17%. A total of 132 genes were annotated in the *R. tanguticum* cp genome, including 87 protein-coding gene, 37 tRNA (25 species), and 8 rRNA (4 species).

A phylogenetic analysis was reconstructed with *R. tanguticum* and 13 other complete cp genomes of species, including 11 species of Caryophyllales and two species of Ericales. *Primula poissonii* and *Vaccinium macrocarpon* (both within Ericales) were set as outgroups (Figure 1). The neighbor-joining (NJ) tree constructed with MEGA X (Kumar et al. 2018). The phylogenetic tree indicated that *R. tanguticum* and *R. palmatum* are more distantly related.

**Figure 1.**
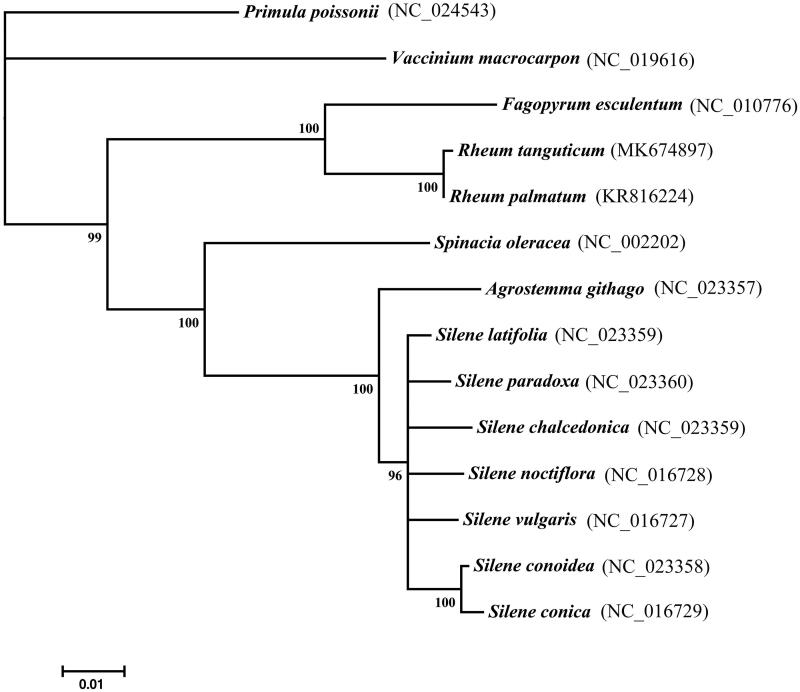
Neighbor-joining tree based on the complete cp genome sequences of 13 taxa, with *Primula poissonii* and *Vaccinium macrocarpon* as outgroup. Shown next to the nodes are bootstrap support values based on 1000 replicates.
